# Identification of Multiple *Blastocystis* Subtypes in Domestic Animals From Colombia Using Amplicon-Based Next Generation Sequencing

**DOI:** 10.3389/fvets.2021.732129

**Published:** 2021-08-24

**Authors:** Adriana Higuera, Giovanny Herrera, Paula Jimenez, Diego García-Corredor, Martin Pulido-Medellín, Diana M. Bulla-Castañeda, Juan Carlos Pinilla, Darwin A. Moreno-Pérez, Jenny G. Maloney, Mónica Santín, Juan David Ramírez

**Affiliations:** ^1^Centro de Investigaciones en Microbiología y Biotecnología-UR (CIMBIUR), Facultad de Ciencias Naturales, Universidad del Rosario, Bogotá, Colombia; ^2^Grupo de Investigación en Medicina Veterinaria y Zootecnia, Facultad de Ciencias Agropecuarias, Universidad Pedagógica y Tecnológica de Colombia, Bogotá, Colombia; ^3^Grupo de Investigación en Ciencias Agropecuarias, Facultad de Ciencias Exactas, Naturales y Agropecuarias, Universidad de Santander, Bucaramanga, Colombia; ^4^Universidad de Ciencias Aplicadas y Ambientales, Bogotá, Colombia; ^5^Environmental Microbial and Food Safety Laboratory, Beltsville Agricultural Research Center (BARC), United States Department of Agriculture (USDA-ARS), Beltsville, MD, United States

**Keywords:** *Blastocystis*, Colombia, farm animals, next generation amplicon sequencing, mixed infection, MinION, subtypes

## Abstract

*Blastocystis* is frequently reported in fecal samples from animals and humans worldwide, and a variety of subtypes (STs) have been observed in wild and domestic animals. In Colombia, few studies have focused on the transmission dynamics and epidemiological importance of *Blastocystis* in animals. In this study, we characterized the frequency and subtypes of *Blastocystis* in fecal samples of domestic animals including pigs, minipigs, cows, dogs, horses, goats, sheep, and llama from three departments of Colombia. Of the 118 fecal samples included in this study 81.4% (*n* = 96) were positive for *Blastocystis* using a PCR that amplifies a fragment of the small subunit ribosomal RNA (*SSU* rRNA) gene. PCR positive samples were sequenced by next generation amplicon sequencing (NGS) to determine subtypes. Eleven subtypes were detected, ten previously reported, ST5 (50.7%), ST10 (47.8%), ST25 (34.3%), ST26 (29.8%), ST21 (22.4%), ST23 (22.4%), ST1 (17.9%), ST14 (16.4%), ST24 (14.9%), ST3 (7.5%), and a novel subtype, named ST32 (3.0%). Mixed infection and/or intra -subtype variations were identified in most of the samples. Novel ST32 was observed in two samples from a goat and a cow. To support novel subtype designation, a MinION based sequencing strategy was used to generate the full-length of the *SSU* rRNA gene. Comparison of full-length nucleotide sequences with those from current valid subtypes supported the designation of ST32. This is the first study in Colombia using NGS to molecularly characterize subtypes of *Blastocystis* in farm animals. A great diversity of subtypes was observed in domestic animals including subtypes previously identified in humans. Additionally, subtype overlap between the different hosts examined in this study were observed. These findings highlight the presence of *Blastocystis* subtypes with zoonotic potential in farm animals indicating that farm animals could play a role in transmission to humans.

## Introduction

*Blastocystis* is a unicellular eukaryote belonging to the phylum Stramenopila that infects the intestine of both humans and animals (1, 2). It has been reported worldwide with estimated prevalence of up to 23.1% in developed countries and 50% in developing countries (3–5). *Blastocystis* is observed in both symptomatic and asymptomatic humans generating controversy about the pathogenic role of this microorganism and its clinical importance (6). Furthermore, *Blastocystis* has been observed in a wide range of both wild and domestic animals including mammals, birds, reptiles and insects (7–10), highlighting a potential risk of zoonotic transmission to humans (1, 8, 11, 12).

A wide genetic diversity of *Blastocystis* has been identified in isolates obtained from birds and mammals based on nucleotide polymorphism at the small subunit ribosomal RNA gene (*SSU* rRNA) that has allowed the establishment of different subtypes (STs) (13, 14). *Blastocystis* subtypes display varying degrees of host specificity (15). At present, there are 31 proposed subtypes, although four of these subtypes are not currently considered valid (13, 14, 16). Of these subtypes, ST1 to ST9 and ST12 have been found in humans (17). ST1 to ST4 are the most common subtypes reported in humans (18), and ST9 has been reported only in humans (19). Subtypes identified in humans have also been reported in domestic and wild animals (12). In farm animals, a combination of zoonotic and enzootic subtypes has been reported (12). In cattle, zoonotic subtypes (ST1-ST7 and ST12) and enzootic subtypes (ST10, ST14, ST17, ST21, ST23-ST26) have been reported (12, 20, 21). Likewise, in small ruminants, zoonotic STs (ST1, ST3, ST4, ST5, ST7) and enzootic STs (ST10, ST12, ST14) are commonly reported (1, 12, 22). In pigs, most studies report zoonotic STs. Eight zoonotic subtypes have been reported (ST1-ST5, ST7), but also enzootic STs (ST10 and ST15) have been reported (12, 23–25). In birds, zoonotic ST6 and ST7 are mainly reported, but other STs have also been detected including additional zoonotic STs (ST1, ST2, ST4-ST5, and ST8) and enzootic STs (ST10, ST13, ST14, ST20, ST24, ST27, ST28, ST29) (1, 12, 26–28). In companion animals, zoonotic STs ST1-ST6 and enzootic ST10 have been reported (12).

Few studies that include molecular characterization in animal hosts in the Americas have been conducted (12). In the United States, ST3-ST5, ST10, ST14, ST17, ST21, and ST23-ST26 were observed in samples from cattle (29), ST5 in swine (23), ST10, ST1, and ST3 in feline and canine samples (30), and ST30 and 31 in white-tailed deer (16). In Mexico, ST4 and ST17 have been detected in rodents (31). In Brazil, ST1 was detected in pigs (25), ST1 and ST3 in dogs (32). ST5-ST7, ST10, ST14, ST24, ST27, and ST28 in captive wild birds (33), and ST6, ST7, ST10, ST14, ST25, and ST29 in chickens (28). In Peru, ST8 was reported in monkeys (34). In Ecuador ST8 was detected in monkeys (35), and in Colombia ST6 was found in birds, ST8 in marsupials, ST4 in howler monkeys and ST1-ST3 were detected in domesticated mammals (26) and ST1 in dogs (36). Previous studies clearly indicate that some STs are shared between humans and animals, showing that zoonotic STs are frequently identified in livestock and companion animals. However, the contribution of animals to human infection has yet to be clarified and studies in this regard are needed.

PCR coupled with Sanger sequencing of a fragment of the *SSU* rRNA gene is commonly used in subtyping studies of *Blastocystis* (37, 38). However, results based on chromatogram analysis of Sanger-sequenced products have shown that mixed infections are common (8, 37, 39). For this reason, if mixed subtype infections are present in a sample, cloning may be required to obtain clear subtype sequences. This process has been used to discern between mixed subtypes present in the same sample, but this process represents additional steps for identification and may miss low-abundance subtypes (8, 39). Next generation amplicon sequencing (NGS) has showed greater sensitivity in detecting mixed subtype infections of *Blastocystis* in humans and animal studies (28, 40–42). For example, NGS found mixed subtype infections in 62.5% of the *Blastocystis*-positive bird samples examined from Brazil (33), 13.7% of human samples from a rural area of Mexico (40) and 51.6% of *Blastocystis*-positive human samples collected from patients with diarrhea in Colombia (42). However, the use of this technique has not been applied to study animal samples from Colombia. Therefore, the aim of this study was to determine the occurrence of *Blastocystis* in farm and companion animals present in different departments of Colombia. NGS was used to conduct molecular characterization and subtyping of *Blastocystis*-positive samples to determine mixed subtypes, presence of low abundance subtypes, and intra-subtype variations. Such information is crucial to understand the epidemiology, animal reservoirs of zoonotic subtypes, and transmission dynamics of *Blastocystis* infections.

## Materials and Methods

### Study Population and Sample Collection

Convenience sampling was conducted to obtain fecal animal samples. One hundred and eighteen fecal samples were collected from healthy and adult domestic animals in the city of Bogotá and the departments of Santander, Boyacá and Cundinamarca in Colombia (Supplementary Figure 1). Domestic animals included: cattle (Holstein breed; *n* = 58), pigs (commercial line SM52xL2PIC; *n* = 3), minipigs (*n* = 35), sheep (*n* = 1), llama (*n* = 1), rabbits (*n* = 3), dogs (*n* = 4), horses (*n* = 11) and goats (*n* = 2). Fecal samples were collected directly from the rectum of each animal. The animals from Bogotá belonged to the veterinary faculty of the University of Applied and Environmental Sciences (UDCA) where they were part of the academic practices of veterinary and zootechnical students. The samples of animals from Boyacá and Cundinamarca corresponded to Holstein cows of dairy production, and the samples from Santander were minipigs kept as pets. The percentages of samples obtained in each department were: Bogotá (38.9%, *n* = 33), Boyacá (23.6%, *n* = 20), Cundinamarca (35.4%, *n* = 30) and Santander (41.3%, *n* = 35). All fecal samples were preserved at −80 °C until DNA extraction without any preservative.

### DNA Extraction and Detection of *Blastocystis* Using PCR

Prior to DNA extraction, approximately 300 μL of each fecal sample was washed with sterile phosphate-buffered saline (PBS 1X) three times. First, 600 ml of PBS were added to each sample, vortexed for 30 s and centrifuged at 3,000 rpm for a minute. The supernatant was discarded. Genomic DNA was extracted from fecal pellet obtained after washes using the Norgen Stool DNA Isolation Kit, Norgen Biotek Corp (Thorold, ON, Canada), following the recommendations of the manufacturer.

To detect *Blastocystis*, a PCR that amplifies a fragment around 119 bp of the *SSU* rRNA gene was performed in a final volume of 9 μL, containing 3.5 μL of GoTaq Green Master Mix (Promega®, Madison, Wisconsin, USA), 2 μL of template DNA, and 1 μL of each primer to obtain a final concentration of 1 μM in the reaction (43). The sequences of the primers used were, FWD F5 (5′-GGTCCGGTGAACACTTTGGATTT-3′) and R F2 (5′-CCTACGGAAACCTTGTTACGACTTCA-3′) (44). The thermal cycling parameters were as follows: 95°C for 5 min; 35 cycles of 95°C for 15 s, 58°C for 1 min and 72°C for 30 s; 72°C for 10 min. Following PCR, the size of each amplicon was assessed using 2% agarose gel electrophoresis followed by staining with SYBR^TM^ Safe (Invitrogen™, Carlsbad, CA, USA).

### Next Generation Amplicon Sequencing and Bioinformatic Analyses

All PCR positive samples were further screened using next generation amplicon sequencing. PCR, library preparation, and sequencing were performed as previously described (45). All prescreened PCR positive samples were further analyzed by PCR using the primers, forward ILMN_Blast505_532F 5′- TCGTCGGCAGCGTCAGATGTGTATAAGAGACAG GGAGGTAGTGACAATAAATC−3 ′ and reverse ILMN_Blast998_1017R 5′- GTCTCGTGGGCTCGGAGATGTGTATAAGAGACAG TGCTTTCGCACTTGTTCATC-3′ (adapter sequences underlined) that amplify a fragment of *ca*. 500 bp of the *SSU* rRNA gene (45). The Illumina 16S Metagenomic Sequencing Library Preparation protocol (Part # 15044223 Rev. B) was used for the library preparation with PCR conditions and preparation of libraries carried out as reported in previous studies (45). Quantification of the libraries was performed using the Quant-iT dsDNA Broad-Range Assay Kit (ThermoFisher, Waltham, MA) on a SpectraMax iD5 (Molecular devices, San Jose, CA) and sequencing was conducted using Illumina MiSeq 600 cycle v3 chemistry (Illumina, San Diego, CA) following the manufacturer's instructions.

Paired-end reads were processed and analyzed with an in-house pipeline that uses the BBTools package v38.82, Bushnell B (2014), BBMap downloaded in http://sourceforge.net/projects/bbmap, VSEARCH v2.15.1 (46), and BLAST + 2.10.1. After removing singletons, clustering and the assignment of centroid sequences to operational taxonomic units (OTU) was performed within each sample at a 98% identity threshold. The unique sequences obtained were assigned to a *Blastocystis* subtype based on the best BLAST result in the GenBank database. All partial sequences generated in this study were deposited in GenBank under the accession numbers MW662458-MW662511.

### PCR Amplification, MinION Sequencing, and Bioinformatic Analysis Used to Generate *Blastocystis* Full-Length SSU rRNA Gene

DNA from a goat (sample #54), was PCR amplified to produce full-length *SSU* rRNA gene sequences to validate novel subtype ST32. A previously described PCR and Nanopore sequencing strategy were used to generate sequences of the approximately 1,800 base pair *SSU* rRNA gene (47) with the following updates. Briefly, a PCR using the MinION-tailed primers SSU-F1 (5′–TTT CTG TTG GTG CTG ATA TTG C AAC CTG GTT GAT CCT GCC AGT AGT C−3′) and SSU-R1 (5′–ACT TGC CTG TCG CTC TAT CTT C TGA TCC TTC TGC AGG TTC ACC TAC G−3′) (adapter sequences underlined) which amplify most eukaryotic organisms' full-length *SSU* rRNA gene was performed using the high-fidelity proofreading polymerase contained in KAPA HiFi HotStart ReadyMix (KAPABioSystems, Cape Town, South Africa). Initial denaturation was performed at 98 °C for 5 min followed by 35 cycles of amplification: 20 s at 98 °C, 45 s at 60 °C, and 90 s at 72 °C. The final extension continued for 5 min. PCR amplicons were purified using a 0.5 × AMPure XP beads (Beckman Coulter, Brea, CA, USA) to sample ratio and quantified on a Qubit fluorometer (ThermoFisher Scientific, Waltham, MA, USA). To prepare the Nanopore sequencing library the Oxford Nanopore Technologies (ONT) SQK-LSK109 and SQK-LSK110 Ligation Sequencing Kits were used following the manufacturer's protocol for PCR Barcoding Amplicons (PBAC12_9112_v110_revB_10Nov2020) and loading guidelines for R9 and R10 flow cells, respectively. The EXP-PBC001 PCR Barcoding Kit (ONT, Oxford, UK) was used in combination with the ligation kits for barcoding each sample. A modification to the barcoding PCR protocol included the use of the KAPA HiFi polymerase described above instead of the NEB LongAmp Taq. Amplicons were purified with 0.5 × XP beads once more, quantified and diluted to ensure 50 or 75 fmol of library in 12 uL was loaded onto an R9 (FLO-MIN106) or R10 flow cell (FLO-MIN111), respectively. R9 flow cells were run aboard an Mk1B MinION and R10 flow cells aboard the Mk1C MinION both using MinKNOW v20.10.06 software.

Basecalling was performed using Guppy v4.4.1 (gpu) and the High Accuracy models available for R9 and R10 flow cells in the following configuration files: dna_r9.4.1_450bps_hac.cfg and dna_r10.3_450bps_hac.cfg. A minimum quality score cut off of 7 was used for filtering low quality reads. FASTQ reads were then length filtered to include only reads between 1,700 and 2,000 nucleotides. Filtered reads were then corrected using canu v2.1.1 and then length filtered again to retain reads between 1,700 and 2,000 nt. Next, MinION PCR adapters were trimmed and only reads with intact forward and reverse eukaryotic primers were retained (bbduk.sh k = 18 restrictleft/right = 150 mm = f edist = 2; BBTools v38.86). Primer orientation was used to ensure all reads were converted to the plus strand before combining them into a single FASTA file. Reads were then clustered using the vsearch –cluster_fast command (vsearch v2.15.1) with a 98% identity threshold and checked for chimeras using the vsearch –uchime_denovo command. The chimera-free clusters were then polished using Racon v1.4.20. The SAM file needed for racon polishing was generated by first extracting non-chimeric reads from pre-clustered reads and mapping them back to the chimera-free clusters using Minimap2 v2.17-r941 and the flags -ax asm5 –secondary = no. Racon-polished consensus sequences were clustered again at 98% identity using the vsearch –cluster_size command and sequences with <10 supporting reads were removed. Another round of polishing was performed using Nanopolish v0.13.2 for R9 reads (nanopolish variants –consensus –min-flanking-sequence = 10 –fix-homopolymers –max-haplotypes = 10,000) or Medaka v1.2.1 for R10 reads (medaka_consensus –m r103_min_high_g360) (medaka). All full-lenght sequences generated in this study were deposited in GenBank under the accession numbers MZ265403-MZ265408.

### Phylogenetic Analysis

The full-length *SSU* rRNA gene nucleotide sequences obtained in this study, appropriate full-length *Blastocystis* reference nucleotide sequences obtained from the reference database found at http://entamoeba.lshtm.ac.uk/blastorefseqs.htm (accessed 5/7/2021), and other full-length sequences of currently accepted STs available in GenBank were included in the analysis. Nucleotide sequences were aligned with the Clustal W algorithm, phylogenetic analysis was performed using the Neighbor-Joining (NJ) method, and genetic distances calculated with the Kimura 2-parameter model using MEGA X (48, 49). The phylogenetic tree was rooted using *Proteromonas lacertae* as an outgroup. A total of 1,951 positions were included in the final dataset that included 70 nucleotide sequences. Bootstrapping with 1,000 replicates was used to determine support for the clades generated. Additionally, evolutionary analysis was conducted to establish divergence between nucleotide sequences (pairwise distance) using the Kimura 2-parameter model in MEGA X.

### Visualization of *Blastocystis* Subtype Frequencies Within and Between Hosts

A bar plot was constructed to observe the inter-subtype variation in each analyzed sample. For this, the percentage of the unique sequences established for each ST was taken into account, and percentages were calculated for each sample. A color for each ST was assigned. The bar plot was constructed considering the hosts of each sequence analyzed and the geographic region of origin of each of the samples.

## Results

### Detection of *Blastocystis*

A total of 118 samples of farm and companion animal fecal samples were tested for the presence of *Blastocystis* by PCR. Overall, 81.4% (*n* = 96) of the 118 samples were positive for *Blastocystis*, of which 27.1% (*n* = 26) were from Bogotá and included cows, horses, dogs, goats, pigs, rabbits, a sheep and a llama, 14.6 % (*n* = 14) were from cows in Boyacá, 23.9% (*n* = 23) were from cows in Cundinamarca and 34.4% (*n* = 33) were from mini pigs in Santander (Table 1).

**Table 1:** Occurrence of *Blastocystis* in domestic animals from different locations in Colombia.

**Location**	**Animal**	**No. of samples collected**	**No. of positive samples by PCR (%)**	**No. of positive samples by sequencing (%)**	No. of samples with mixed infection^*^	**Subtypes detected**
Bogotá	Cow	8	8 (100)	8 (100)	8 (100)	ST10, ST14, ST21, ST23 -ST26, ST32
	Dog	4	2 (50)	1 (25)	1 (100)	ST23, ST24
	Goat	2	2 (100)	2 (100)	2 (100)	ST10, ST14, ST21, ST23-ST26, ST32
	Horse	11	6 (54.5)	2 (18.2)	1 (50)	ST10, ST14, ST24
	Llama	1	1 (100)	1 (100)	1 (100)	ST10, ST21, ST23-ST25
	Sheep	1	1 (100)	1 (100)	1 (100)	ST10, ST14, ST21, ST23, ST24, ST26
	Pig	3	3 (100)	3 (100)	0	ST5
	Rabbit	3	3 (100)	0	0	-
Boyacá	Cow	20	14 (70)	5 (25)	4 (80)	ST5, ST10, ST21, ST23, ST25, ST26
Cundinamarca	Cow	30	23 (76.7)	14 (46.7)	14 (100)	ST10, ST14, ST21, ST23, ST25, ST26
Santander	Minipig	35	33 (94.3)	30 (85.7)	12 (40)	ST1, ST3, ST5
Total		118	96 (81.4)	67 (56.8)	44 (65.7)	

* *Percentage of samples with diffferent subtype combinations in mixed infections detected with NGS*.

### Subtypes of *Blastocystis*

Of the 96 samples positive by the PCR, 67 produced *Blastocystis* sequences using NGS. There were 53 unique *Blastocystis* sequences detected among the 67 samples analyzed by NGS, with an average of 3.6 ± 0.7 unique sequences per sample and an average abundance of 71,585 ± 11,534 reads per sample. Unique sequences corresponded to eleven STs, 10 previously reported (ST1, ST3, ST5, ST10, ST14, ST21, ST23, ST24, ST25, ST26) and a novel subtype (named ST32). The most common STs were: ST5 50.7% (*n* = 34) and ST10 47.8% (*n* = 32) followed by ST25 34.3% (*n* = 23), ST26 29.8% (*n* = 20), ST21 22.4% (*n* = 15), ST23 22.4% (*n* = 15), ST1 17.9% (*n* = 12), ST14 16.4% (*n* = 11), ST24 14.9% (*n* = 10), ST3 7.5% (*n* = 5) and ST32 3.0% (*n* = 2).

Subtypes identified in each host are summarized in Table 1. In ruminants a wide genetic diversity was observed with nine STs identified in cattle (ST5, ST10, ST14, ST21, ST23 ST24, ST25, ST26, and ST32), eight in goats (ST10, ST14, ST21, ST23, ST24, ST25, ST26, and ST32) and six in sheep (ST10, ST14, ST21, ST23, ST24, and ST26) (Table 1). In the only *Blastocystis*-positive llama, five STs (ST10, ST21, ST23, ST24, and ST25) were identified, while three STs (ST10, ST14, and ST24) were identified in horses. In pigs and minipigs, one (ST5) and three (ST1, ST3, and ST5) subtypes were identified, respectively. Two subtypes were identified in dogs (ST23 and ST24).

ST10 had the greatest intra-subtype variation, with 16 unique sequences (16/53; 30.2%), followed by ST1 (8/53; 15.1%), ST26 (6/53; 11.3%), ST14 and ST5 (each 5/53; 9.4%), ST25 (4/53; 7.5%), ST24 (3/53; 5.7%), ST3 and ST21 (each 2/53; 3.8%), and ST23 and ST32 (each 1/53; 1.9%). Intra-subtype variation was commonly observed within the same sample (Supplementary Table 1). Up to 12 different unique sequences were observed in a single sample (sample #40).

**Figure 1:**
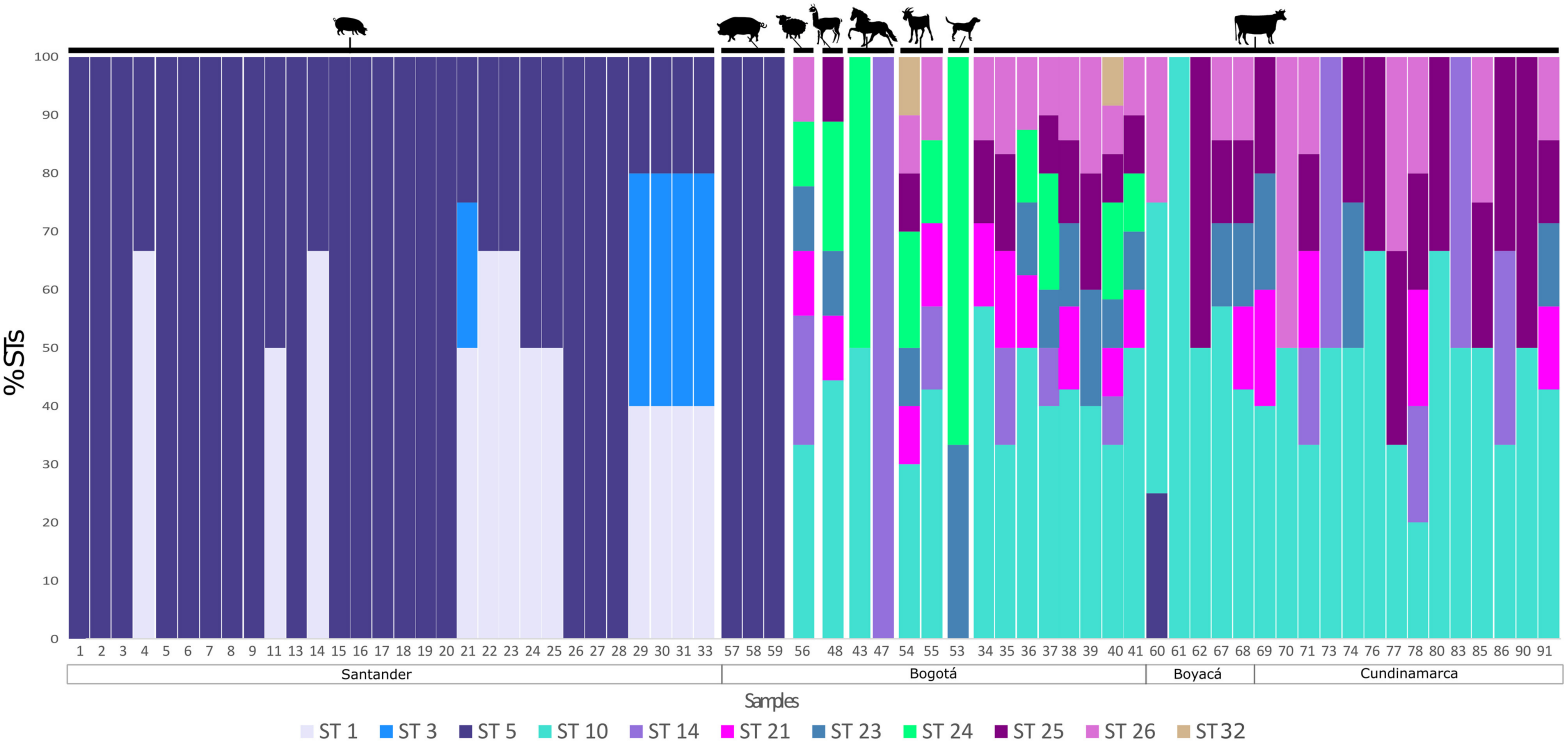
Detection of single and mixed infections in animals. The percentage of each ST detected is shown by sample. Each ST is highlighted in a different color. The figure is organized by host and location.

Figure 1 shows multiple STs found within the same host and in the same sample. The corresponding number of each sample is shown in the x axis. Also, the region where each sample was collected is shown. The numbers on the y axis represent the frequency of each ST found for each sample. From minipigs, 18/30 samples showed mono-infections with ST5, but in 7/30 samples ST1 and ST5 were found and 5/30 samples had infections with ST1, ST3, and ST5. All three pig samples contained ST5. In the case of cattle, ST10 was the most commonly found subtype, but mixed infections were evident in the most of samples. Among all animals, in Bogotá, we found ST24 and ST32 that are absent in samples from Boyacá and Cundinamarca. Samples from sheep, llama, and goat also showed a greater inter-subtype variation, while in the samples of horse mono-infections were observed and in the sample of dog just two STs were found. The novel subtype (ST32) was detected in samples 40 and 54 from a cow and a goat, respectively.

### MinION Sequencing and Phylogenetic Support for Novel Subtype

To confirm the validity of the novel subtype according to recently proposed guidelines (13), the near full-length nucleotide sequence of the SSU rRNA gene should be obtained. A MinION long read sequencing strategy was applied to one goat sample which contained the novel subtype (sample #54). A full-length sequence of the *SSU* rRNA gene was successfully obtained for the novel subtype. Multiple subtypes were present in sample number 54, and five additional full-length nucleotide sequences were also obtained for five other subtypes, ST10, ST21, ST23, ST24, and ST26.

**Figure 2:**
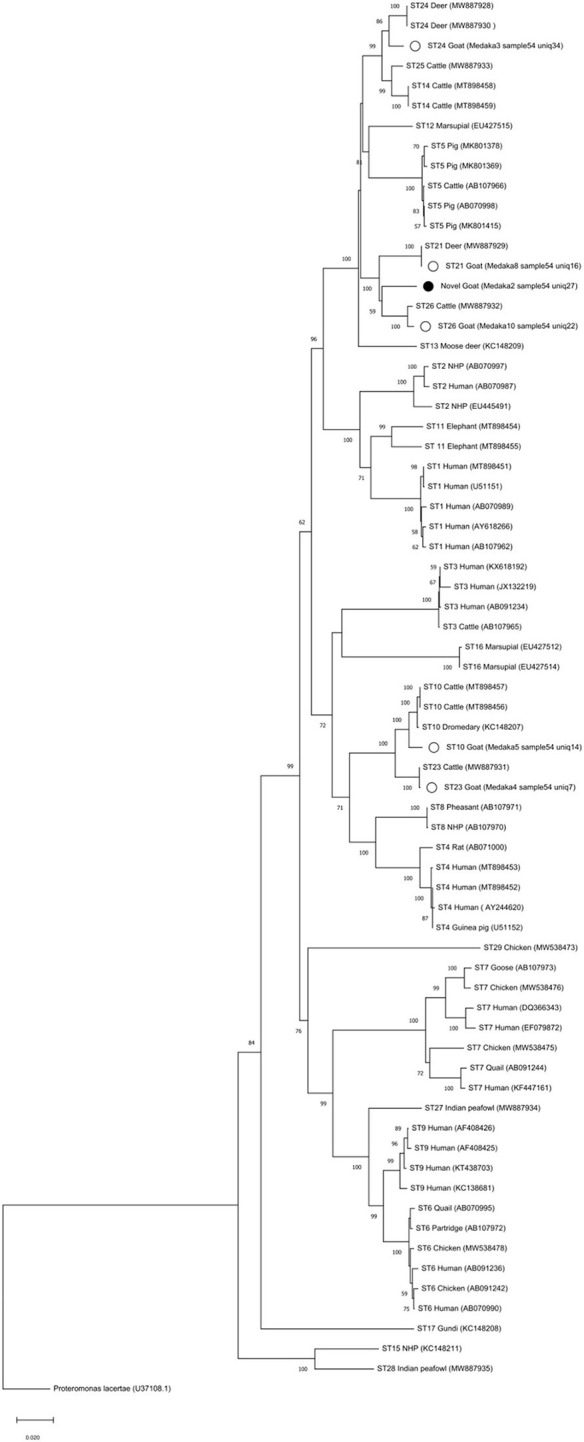
Phylogenetic reconstruction with MinION sequences. The full-length *SSU* rRNA gene nucleotide sequences obtained in this study were aligned with *Blastocystis* reference nucleotide sequences (accession numbers in parenthesis). Nucleotide sequences were aligned with the Clustal W algorithm and phylogenetic analysis was performed using the Neighbor-Joining (NJ) method and genetic distances calculated with the Kimura 2-parameter model using MEGA X (48, 49). Bootstrapping with 1,000 replicates was used to support the clades. The bootstrap number is shown on each node. *Proteromonas lacertae* was used as an outgroup. White circles show the samples with full-length obtained in this study and the black circle is showing the novel subtype found, called ST32.

Phylogenetic analysis of full-length sequences by NJ method showed that ST32 clusters with ST21 and ST26 (Figure 2). Full-length sequences for ST10, ST21, ST23, ST24, and ST26 generated in this study all cluster with corresponding full-length sequences available in GenBank. Pairwise comparison was used to evaluate percent similarity between ST32 and the 25 currently available valid subtypes (ST1-ST17, ST21, ST23-ST29) using full-length sequences (Supplementary Table 2). The highest percentage of sequence similarity for ST32 was 96% with ST21 and ST26.

## Discussion

In the Americas, the number of studies in humans and animals related to *Blastocystis* infection has been increasing which has contributed important data on the prevalence and variability of this microorganism (50–53). However, studies in South America about *Blastocystis* specifically in animals are scarce. In some South American countries, there are studies reporting different *Blastocystis* STs in humans and their relationship with symptomatic patients (54–58). Countries from the Americas where animal samples have been tested from *Blastocystis* include United States (16, 23, 29, 59), Brazil (25), Mexico (31), Ecuador (35), Peru (34) and Colombia (26). In those studies, a relative specific association of STs with hosts has been found. For this reason, it is imperative to increase the available data on the biology of this microorganism, its epidemiological and genetic characteristics, including subtyping and possible relationships with different hosts, to clarify the transmission dynamics which could involve domestic and wild animals as sources of contaminated water (60).

In our study, we found a high frequency of farm animals infected with *Blastocystis* (Table 1). Of the 118 samples screened, 96 (81.4%) were positive for *Blastocystis* by PCR. Cows and pigs were the most studied animals and had high frequency of infection, 77.6 and 68.4%, respectively. These findings were in concordance with previous studies in which *Blastocystis* was a common parasite of these hosts. A wide range of prevalence in cows has been reported in many countries, 1.8% in Spain (61), 71% in French (8), 80% in Colombia (26), and 100% in Indonesia (21), where different factors may be influencing the detection of *Blastocystis* (12). *Blastocystis* is highly prevalent in pigs worldwide with prevalence ranging from 8.3% in Philippines (62), 45.2% in Cambodia, 76.7% in Southeast Queensland (24), 77% in Brazil (63), and up to 100% in Australia and Vietnam (1, 64). Like in cows, the high prevalence in pigs could be related with the age, gender and immune status of the host, but current data point to age as the principal factor linked to prevalence (12). In our study, few samples of llama, sheep and goats were collected (1, 1, and 2, respectively), but all samples were *Blastocystis*-positive. These results are similar to other studies which had a limited number of samples from these hosts but have shown prevalence ranges including 5.5–63.6% in sheep in China and United Arab Emirates (65, 66). In goats, reported prevalence include 0.3% in China (67), 30% in Malaysia (68), and 94.7% in Thailand (69) with highest prevalence in older specimens. There is only one published report of *Blastocystis* from a llama which included only one fecal sample from a circus animal (70). The sample was analyzed and found positive for *Blastocystis* by microscopy. We also obtained samples from companion animals in this study, with samples from four dogs and eleven horses. *Blastocystis* was observed in 50% of samples from dogs and 54.5% of samples from horses. Studies in equine are scarce, just one study reported one positive sample from eight horses analyzed in Thailand (71). In the case of dogs, there are some studies showing prevalence ranges including 1.3% in Cambodia (72), 18.8% in Iran (73), 37% in Colombia (26), and 100% in Australia (74). It is probable the differences in prevalence are related with living conditions that impact dog health, management, and hygiene (12). The last group of animals that we tested for *Blastocystis* were three rabbits, of which 100% were positive, but it is important to note the small number of samples. In two studies reported from China, a low prevalence was found (1–3.3%) using a larger sample size (65, 75), so it would be important to verify the high prevalence detected in our rabbit samples through future studies which increase the number of specimens analyzed.

In Colombia and in most South American countries, few studies have sought to determine the STs circulating in animals. However, current data indicate that animals may be hosts to potentially zoonotic subtypes and could play a role in the transmission of *Blastocystis* to humans (18, 27, 76, 77). In this study, only ST5 was detected in the three pigs from Bogotá housed for academic purposes in the University of Applied and Environmental Sciences, while three subtypes (ST1, ST3, and ST5) were identified in minipigs from Santander (Figure 1). Minipigs live as pets in close contact with humans and the presence of ST1 and ST3 may be related to this close contact with humans as these two subtypes are among the most frequently reported in humans (76, 78–81) (Figure 1). In contrast, ST5 is less commonly observed in human samples and is mainly identified in swine (12, 19). Similarly, a review in Asia that included several animals, with cattle and poultry being the most studied, showed that ST1-ST10, ST12 and ST14 were detected, and ST5 was the most widespread subtype in the animals studied (10). Furthermore, many of the subtypes reported were those more frequently found in humans indicating probable zoonotic transmission (10). In our case, it would be important to include samples from the keepers of these animals to support the occurrence of zoonotic transmission. Therefore, better monitoring is necessary, both of the STs found in animals close to human populations as well as of the STs present in humans that are in close contact with animals and even of those human populations where there is a greater possibility to come into contact with wild animals whose frequency and diversity of *Blastocystis* have yet to be explored. Such studies would establish a better picture of the zoonotic potential and transmission dynamics of *Blastocystis*. For example, in the case of a study in Malaysia, it was determined that people who are in close contact with animals, like animal handlers, are more likely to become infected with *Blastocystis* since 41% of the animal handlers were positive for this parasite (11). Similar results were found in another study conducted in animals from French zoos where they found shared subtypes between some animals and animal handlers (8). In mono-subtype infections it is possible to infer a common source of contamination. In the case of mixed subtype infections, subtyping allows for hypotheses about the possible routes of transmission between different hosts. Clearly accurate subtyping is key to understand transmission and to develop prevention and control strategies in the future.

In the present study, subtyping was performed using NGS as previously reported in Mexico using human stool samples (40), in Brazil in captive wild birds and chickens (28, 33), in the United States in cattle and deer (16, 29), in Spain in wild carnivores (41), in Italy in wild boars and pigs (82). NGS has been compared to Sanger sequencing and cloning and allowing to identify the presence of mixed infections more easily without costly processes such as cloning of PCR products and subsequent sequencing of multiple clones (45). Because in samples from feces mixed infections are common (8, 83, 84), it is important to deploy NGS tools in studies of *Blastocystis* subtype diversity to have a better picture of STs present within a host. The usefulness of NGS in detecting mixed infections has also been demonstrated in other organisms such as *Trypanosoma cruzi* and *Trypanosoma rangeli* where NGS successfully detected co-infections of these two parasites in infected triatomines (85) and in triatomine food sources (86). NGS has aided in the determination of the diversity of low-density *Plasmodium falciparum* infections (87). NGS has also been used in detection of mixed assemblages and intra-assemblage variation in *Giardia duodenalis* infections (88). Using NGS we detected great variability in subtypes among *Blastocystis-*positive samples from farm animals, mixed infections inter e intra subtype and a novel subtype, which was confirmed with the full-length sequence. Among the most prevalent STs, we found ST10 and ST14 as previously reported in cattle (1, 59, 89). These findings expand the subtype diversity of *Blastocystis* in cattle from Colombia where only ST1 and ST3 have been reported (26) whereas we found ST10, ST21, ST23, ST24, ST25, ST26, and ST32. Also, they tested dog samples and reported just ST2, but we found ST23 and ST24. ST5 was found in high frequency in samples from pigs and minipigs similar to previous studies from pigs (10, 15, 24). ST10, ST14 and ST24 were found in two samples of horses contrasting with the ST1 found using a partial sequence of the SSU-rDNA gene (90).

Inter-ST variation and intra-ST variation within the same sample were detected. Unique sequences with differences between 2 and 11% were identified that correspond to the same ST in accordance with another study where different unique sequences were detected in the same subtype (45). This variation was detected mainly in ST5, ST10, ST14, and ST26 in the samples from Bogotá, Boyacá and Cundinamarca, which curiously exclude animals that live with humans. The only animals used as pets were the minipigs, the rest of animals are only in contact with animal handlers and veterinary and zootechnical students. Our results demonstrate the great variation of subtypes presents in farm animals in Colombia, but the role of this diversity in animal health is still unknown. Also, it is not known if these subtypes can complete their life cycle within the evaluated hosts and be transmitted to humans or other animals. The frequency of mixed infections in animals could be due to socioeconomic conditions in some regions of the country and poor hygienic practices that produce high rates of intestinal parasite infection (43), compared to developed countries, but more studies exploring mixed infections are necessary to reach conclusions. On the other hand, the importance of the mixed infections could also be related with the effect of this variability over the microbiota. For example, one study in humans evaluated the co-occurrence of *Blastocystis* with a bacterial pathogen *Clostridioides difficile*, where the bacterial family with the highest abundance detected in the samples with mixed *Blastocystis* infections was mainly Pseudomonadaceae, while in the samples with a single *Blastocystis* subtype there was a greater abundance of potentially beneficial families such as Prevotellaceae (42). Therefore, our findings highlight the need to use techniques such as NGS that allow us to determine STs and/or unique sequences of other STs that are found in lower abundance in an analyzed sample and that may be important to clarify the genetic variability present in *Blastocystis* given the different hosts that may be included in its transmission cycle, mainly among those that live in close spaces, share the same green areas and whose caretakers are usually the same.

A new ST was found in two samples in this study, from a cow and a goat. To validate its designation as a novel ST, we obtained the complete sequence of the *SSU* rRNA gene using a MinIOn sequencing strategy (47). In addition to the full-length of the *SSU* rRNA gene nucleotide sequence of the novel ST, five additional full-length nucleotide sequences were obtained (ST10, ST21, ST23, ST24, and ST26) as the goat was infected with multiples STs and that diversity was captured using the MinIOn sequencing strategy too. The phylogenetic analysis of full-length sequences including all valid STs, showed that novel ST clustered with ST21 and ST26 (Figure 2). Pairwise analysis showed that closest STs were ST21 and ST26, with a similarity of 96% (Supplementary Table 2). Therefore, the novel ST complied with the current established criteria for the determination of novel STs (13) and was named ST32. Our results highlight the importance of obtaining the full-length sequences of the *SSU* rDNA gene of *Blastocystis* to verify potential novel STs that should meet the 4% of divergence and to use in phylogenetic analyses as these results are more robust in comparison to partial region analyses.

In conclusion, this is the first study in Colombia in farm and companion animals to use NGS to subtype *Blastocystis*. Multiple STs were present in most samples and intra-subtype variation was also common. Our findings highlight the importance of using a method such as NGS that allows for identification of genetic variation within a sample and that allows identification of STs present in low abundance. This information is important to understand transmission dynamics of this parasite and the evaluation of the zoonotic potential of the STs present in the sampled animals. It is necessary to evaluate other groups of animals that could be possible reservoirs of *Blastocystis* and to expand number of samples and the regions of Colombia studied. Incorporating samples from humans who are in close contact with animals and other possible reservoirs such as water sources consumed by both animals and humans is also necessary to determine the transmission dynamics of *Blastocystis*.

## Data Availability Statement

The datasets presented in this study can be found in online repositories. The names of the repository/repositories and accession number(s) can be found below: https://www.ncbi.nlm.nih.gov/genbank/, MZ265403-MZ265408.

## Ethics Statement

The animal study was reviewed and approved by Universidad del Rosario. Written informed consent was obtained from the owners for the participation of their animals in this study.

## Author Contributions

AH: extraction, amplification and analysis processes of the evaluated samples, and writing the manuscript. GH: collection the samples from Bogotá and map design. PJ and DM-P: collection the samples from Bogotá. DG-C: collection of samples from Boyacá and Cundinamarca. MP-M, DB-C, and JP: collection of samples from Santander. JM and MS: DNA sequencing, bioinformatic analyses, and correction of the manuscript. JR: contributed to data analysis and revision of the manuscript. All authors contributed to the article and approved the submitted version.

## Conflict of Interest

The authors declare that the research was conducted in the absence of any commercial or financial relationships that could be construed as a potential conflict of interest.

## Publisher's Note

All claims expressed in this article are solely those of the authors and do not necessarily represent those of their affiliated organizations, or those of the publisher, the editors and the reviewers. Any product that may be evaluated in this article, or claim that may be made by its manufacturer, is not guaranteed or endorsed by the publisher.

## Supplementary Material

Supplementary Figure 1 Geographic locations of regions in which samples were collected. Departments of Colombia sampled are indicated in the color blue. Orange circles indicate the exact locations of sampling areas. In the legend on the right, the cities/municipalities sampled in each department are shown.

Supplementary Table 1 Data of partial sequences obtained, hosts, subtypes and geographic region where samples were collected.

Supplementary Table 2 Estimates of Evolutionary Divergence between Sequences.

## References

[1] Alfellani MA Taner-Mulla D Jacob AS Imeede CA Yoshikawa H Stensvold CR. Genetic diversity of Blastocystis in livestock and zoo animals. Protist. (2013) 164:497–509. 10.1016/j.protis.2013.05.003 23770574

[2] Villalobos G Orozco-Mosqueda GE Lopez-Perez M Lopez-Escamilla E Córdoba-Aguilar A Rangel-Gamboa L. Suitability of internal transcribed spacers (ITS) as markers for the population genetic structure of blastocystis spp. Parasit Vect. (2014) 7:1–9. 10.1186/s13071-014-0461-2 25274498 PMC4203911

[3] Stensvold CR Christiansen DB Olsen KEP Nielsen HV. Blastocystis sp. subtype 4 is common in Danish Blastocystis-positive patients presenting with acute diarrhea. Ame J Trop Med Hyg. (2011) 84:883–5. 10.4269/ajtmh.2011.11-0005 21633023 PMC3110361

[4] El Safadi D Gaayeb L Meloni D Cian A Poirier P Wawrzyniak I. Children of senegal river basin show the highest prevalence of blastocystis sp. ever observed worldwide. BMC Infect Dis. (2014) 14:1–11. 10.1186/1471-2334-14-164 24666632 PMC3987649

[5] Osman M El Safadi D Cian A Benamrouz S Nourrisson C Poirier P. Prevalence and risk factors for intestinal protozoan infections with cryptosporidium, giardia, blastocystis and dientamoeba among schoolchildren in tripoli, lebanon. PLoS Negl Trop Dis. (2016) 10:e0004496. 10.1371/journal.pntd.0004496 26974335 PMC4790957

[6] Stensvold CR Nielsen HV Mølbak K Smith HV. Pursuing the clinical significance of blastocystis–diagnostic limitations. Trends Parasitol. (2009) 25:23–9. 10.1016/j.pt.2008.09.010 19013108

[7] Parkar U Traub RJ Vitali S Elliot A Levecke B Robertson I. Molecular characterization of blastocystis isolates from zoo animals and their animal-keepers. Vet Parasitol. (2010) 169:8–17. 10.1016/j.vetpar.2009.12.032 20089360

[8] Cian A El Safadi D Osman M Moriniere R Gantois N Benamrouz-Vanneste S. Molecular epidemiology of blastocystis sp. In various animal groups from two French zoos and evaluation of potential zoonotic risk. PLoS ONE. (2017) 12:e0169659. 10.1371/journal.pone.0169659 28060901 PMC5217969

[9] Ning CQ Hu ZH Chen JH Ai L Tian LG. Epidemiology of blastocystis infection from 1990 to 2019 in China. Infect Dis Poverty. (2020) 9:1–14. 10.1186/s40249-020-00779-z 33380335 PMC7772921

[10] Rauff-Adedotun AA Zain SNM Haziqah MTF. Current status of blastocystis sp. in animals from Southeast Asia: a review. Parasitol Res. (2020) 119:1–12. 10.1007/s00436-020-06828-8 32951145 PMC7502158

[11] Salim HR Kumar GS Vellayan S Mak JW Anuar AK Init I. Blastocystis in animal handlers. Parasitol Res. (1999) 85:1032–33. 10.1007/s004360050677 10599928

[12] Hublin JS Maloney JG Santin M. Blastocystis in domesticated and wild mammals and birds. Res Vet Sci. (2020) 135:260–82. 10.1016/j.rvsc.2020.09.031 33046256

[13] Stensvold CR Clark CG. Pre-empting pandora's box: blastocystis subtypes revisited. Trends Parasitol. (2020) 36:229–32. 10.1016/j.pt.2019.12.009 32001133

[14] Maloney JG Santin M. Mind the gap: new full-length sequences of blastocystis subtypes generated via oxford nanopore minion sequencing allow for comparisons between full-length and partial sequences of the small subunit of the ribosomal RNA gene. Microorganisms. (2021) 9:997. 10.3390/microorganisms9050997 34063045 PMC8147991

[15] Jiménez PA Jaimes JE Ramírez JD. A summary of blastocystis subtypes in North and South America. Parasit Vect. (2019) 12:1–9. 10.1186/s13071-019-3641-2 31358042 PMC6664531

[16] Maloney JG Jang Y Molokin A George NS Santin M. Wide genetic diversity of Blastocystis in white-tailed deer (Odocoileus virginianus) from Maryland, US. Microorganisms A. (2021) 9:1343. 10.3390/microorganisms9061343 34205799 PMC8233720

[17] Ramírez JD Sánchez A Hernández C Flórez C Bernal MC Giraldo JC. Geographic distribution of human blastocystis subtypes in South America. Infect Genet Evol. (2016) 41:32–5. 10.1016/j.meegid.2016.03.017 27034056

[18] Stensvold CR Alfellani MA Nørskov-Lauritsen S Prip K Victory EL Maddox C. Subtype distribution of blastocystis isolates from synanthropic and zoo animals and identification of a new subtype. Int J Parasitol. (2009) 39:473–9. 10.1016/j.ijpara.2008.07.006 18755193

[19] Andersen LOB Stensvold CR. Blastocystis in health and disease: are we moving from a clinical to a public health perspective?. J Clin Microbiol. (2016) 54:524–8. 10.1128/JCM.02520-15 26677249 PMC4767957

[20] Santín M Gómez-Muñoz MT Solano-Aguilar G Fayer R. Development of a new PCR protocol to detect and subtype blastocystis spp. From humans and animals. Parasitol Res. (2011) 109:205–12. 10.1007/s00436-010-2244-9 21210149

[21] Suwanti LT Susana Y Hastutiek P Suprihati E Lastuti NDR. Blastocystis spp. Subtype 10 infected beef cattle in Kamal and Socah, Bangkalan, Madura, Indonesia. Vet World. (2020) 13:231. 10.14202/vetworld.2020.231-237 32255963 PMC7096301

[22] Song JK Yin YL Yuan YJ Tang H Ren GJ Zhang HJ. First genotyping of blastocystis sp. In dairy, meat, and cashmere goats in northwestern China. Acta Trop. (2017) 176:277–82. 10.1016/j.actatropica.2017.08.028 28864325

[23] Fayer R Elsasser T Gould R Solano G Urban J Santin M. Blastocystis tropism in the pig intestine. Parasitol Res. (2014) 113:1465–72. 10.1007/s00436-014-3787-y 24535732

[24] Wang W Owen H Traub RJ Cuttell L Inpankaew T Bielefeldt-Ohmann H. Molecular epidemiology of blastocystis in pigs and their in-contact humans in southeast queensland, Australia, and Cambodia. Vet Parasitol. (2014) 203:264–9. 10.1016/j.vetpar.2014.04.006 24785292

[25] Moura RGF Oliveira-Silva MBD Pedrosa AL Nascentes GAN Cabrine-Santos M. Occurrence of blastocystis spp. In domestic animals in Triângulo Mineiro area of Brazil. Rev Soc Bras Med Trop. (2018) 51:240–3. 10.1590/0037-8682-0484-2016 29768563

[26] Ramírez JD Sánchez LV Bautista DC Corredor AF Flórez AC Stensvold CR. Blastocystis subtypes detected in humans and animals from Colombia. Infect Genet Evol. (2014) 22:223–8. 10.1016/j.meegid.2013.07.020 23886615

[27] Greige S El Safadi D Bécu N Gantois N Pereira B Chabé M. Prevalence and subtype distribution of blastocystis sp. Isolates from poultry in Lebanon and evidence of zoonotic potential. Parasit Vectors. (2018) 11:1–10. 10.1186/s13071-018-2975-5 29973261 PMC6030734

[28] Maloney JG da Cunha MJ Molokin A Cury MC Santin M. Next-generation sequencing reveals wide genetic diversity of blastocystis subtypes in chickens including potentially zoonotic subtypes. Parasitol Res. (2021) 120:2219–31. 10.1007/s00436-021-07170-3 33904983

[29] Maloney JG Lombard JE Urie NJ Shivley CB Santin M. Zoonotic and genetically diverse subtypes of blastocystis in US pre-weaned dairy heifer calves. Parasitol Res. (2019) 118:575–82. 10.1007/s00436-018-6149-3 30483890

[30] Ruaux CG Stang BV. Prevalence of blastocystis in shelter-resident and client-owned companion animals in the US Pacific Northwest. PLoS ONE. (2014) 9:e107496. 10.1371/journal.pone.0107496 25226285 PMC4166454

[31] Martinez-Hernandez F Martinez-Ibarra JA Lopez-Escamilla E Villanueva-Garcia C Muñoz-Garcia CI Rendon-Franco E. Molecular genotyping of blastocystis spp. In wild mammals from Mexico. Parasitol Res. (2020) 119:97–104. 10.1007/s00436-019-06530-4 31735993

[32] David ÉB Guimarães S de Oliveira AP de Oliveira-Sequeira TCG Bittencourt GN Nardi ARM. Molecular characterization of intestinal protozoa in two poor communities in the State of São Paulo, Brazil. Parasit Vectors. (2015) 8:1–12. 10.1186/s13071-015-0714-8 25889093 PMC4335703

[33] Maloney JG Molokin A da Cunha MJR Cury MC Santin M. Blastocystis subtype distribution in domestic and captive wild bird species from Brazil using next generation amplicon sequencing. Parasite Epidemiol Control. (2020) 9:e00138. 10.1016/j.parepi.2020.e00138 32021915 PMC6995250

[34] Helenbrook WD Whipps CM. Molecular characterization of blastocystis in captive and free-ranging new world primates, platyrrhini. Acta Parasitol. (2021). 10.1007/s11686-021-00397-1. [Epub ahead of print]. 33914238

[35] Helenbrook WD Shields WM Whipps CM. Characterization of blastocystis species infection in humans and mantled howler monkeys, alouatta palliata aequatorialis, living in close proximity to one another. Parasitol Res. (2015) 114:2517–25. 10.1007/s00436-015-4451-x 25859926

[36] Villamizar X Higuera A Herrera G Vasquez-ALR Buitron L Muñoz LM. Molecular and descriptive epidemiology of intestinal protozoan parasites of children and their pets in Cauca, Colombia: a cross-sectional study. BMC Infect Dis. (2019) 19:1–11. 10.1186/s12879-019-3810-0 30808303 PMC6390308

[37] Scanlan PD Stensvold CR Cotter PD. Development and application of a blastocystis subtype-specific PCR assay reveals that mixed-subtype infections are common in a healthy human population. Appl Environ Microbiol. (2015) 81:4071–6. 10.1128/AEM.00520-15 25841010 PMC4524157

[38] Clark CG van der Giezen M Alfellani MA Stensvold CR. Recent developments in blastocystis research. Adv Parasitol. (2013) 82:1–32. 10.1016/B978-0-12-407706-5.00001-0 23548084

[39] Meloni D Poirier P Mantini C Noël C Gantois N Wawrzyniak I. Mixed human intra-and inter-subtype infections with the parasite blastocystis sp. Parasitol Int. (2012) 61:719–22. 10.1016/j.parint.2012.05.012 22659011

[40] Rojas-Velázquez L Maloney JG Molokin A Morán P Serrano-Vázquez A González E. Use of next-generation amplicon sequencing to study blastocystis genetic diversity in a rural human population from mexico. Parasit Vectors. (2019) 12:1–9. 10.1186/s13071-019-3814-z 31775832 PMC6882168

[41] Calero-Bernal R Santín M Maloney JG Martín-Pérez M Habela MA Fernández-García JL. Blastocystis sp. Subtype diversity in wild carnivore species from Spain. J Eukaryot Microbiol. (2020) 67:273–8. 10.1111/jeu.12772 31691450

[42] Vega L Herrera G Muñoz M Patarroyo MA Maloney JG Santín M. Gut microbiota profiles in diarrheic patients with co-occurrence of clostridioides difficile and blastocystis. PLoS ONE. (2021) 16:e0248185. 10.1371/journal.pone.0248185 33725006 PMC7963057

[43] Higuera A Villamizar X Herrera G Giraldo JC Vasquez-ALR Urbano P. Molecular detection and genotyping of intestinal protozoa from different biogeographical regions of Colombia. PeerJ. (2020) 8:e8554. 10.7717/peerj.8554 32195042 PMC7067185

[44] Stensvold CR Ahmed UN Andersen LOB Nielsen HV. Development and evaluation of a genus-specific, probe-based, internal-process-controlled real-time PCR assay for sensitive and specific detection of blastocystis spp. J Clin Microbiol. (2012) 50:1847–51. 10.1128/JCM.00007-12 22422846 PMC3372105

[45] Maloney JG Molokin A Santin M. Next generation amplicon sequencing improves detection of blastocystis mixed subtype infections. Infect Genet Evol. (2019) 73:119–25. 10.1016/j.meegid.2019.04.013 31026606

[46] Rognes T Flouri T Nichols B Quince C Mahé F. VSEARCH: a versatile open source tool for metagenomics. PeerJ. (2016) 4:e2584. 10.7717/peerj.2584 27781170 PMC5075697

[47] Maloney JG Molokin A Santin M. Use of Oxford nanopore MinION to generate full-length sequences of the blastocystis small subunit (SSU) rRNA gene. Parasit Vect. (2020) 13:1–8. 10.1186/s13071-020-04484-6 33239096 PMC7687777

[48] Kimura M. A simple method for estimating evolutionary rates of base substitutions through comparative studies of nucleotide sequences. J Mol Evol. (1980) 16:111–20. 10.1007/BF01731581 7463489

[49] Kumar S Stecher G Li M Knyaz C Tamura K. MEGA X: molecular evolutionary genetics analysis across computing platforms. Mol Biol Evol. (2018) 35:1547. 10.1093/molbev/msy096 29722887 PMC5967553

[50] Jones MS Whipps CM Ganac RD Hudson NR Boroom K. Association of blastocystis subtype 3 and 1 with patients from an Oregon community presenting with chronic gastrointestinal illness. Parasitol Res. (2009) 104:341–5. 10.1007/s00436-008-1198-7 18923844

[51] Whipps CM Boorom K Bermudez LE Kent ML. Molecular characterization of blastocystis species in Oregon identifies multiple subtypes. Parasitol Res. (2010) 106:827–32. 10.1007/s00436-010-1739-8 20127113

[52] Scanlan PD Knight R Song SJ Ackermann G Cotter PD. Prevalence and genetic diversity of blastocystis in family units living in the United States. Infect Genet Evol. (2016) 45:95–7. 10.1016/j.meegid.2016.08.018 27545648

[53] Nash AK Auchtung TA Wong MC Smith DP Gesell JR Ross MC. The gut mycobiome of the human microbiome project healthy cohort. Microbiome. (2017) 5:1–13. 10.1186/s40168-017-0373-4 29178920 PMC5702186

[54] Casero RD Mongi F Sánchez A Ramírez JD. Blastocystis and urticaria: examination of subtypes and morphotypes in an unusual clinical manifestation. Acta trop. (2015) 148:156–61. 10.1016/j.actatropica.2015.05.004 25976414

[55] Ramírez JD Flórez C Olivera M Bernal MC Giraldo JC. Blastocystis subtyping and its association with intestinal parasites in children from different geographical regions of Colombia. PLoS ONE. (2017) 12:e0172586. 10.1371/journal.pone.0172586 28222192 PMC5319748

[56] Rojas-Velázquez L Morán P Serrano-Vázquez A Fernández LD Pérez-Juárez H Poot-Hernández AC. Genetic diversity and distribution of blastocystis subtype 3 in human populations, with special reference to a rural population in central Mexico. BioMed Res Int. (2018) 2018:3916263. 10.1155/2018/3916263 29744356 PMC5878905

[57] de Melo GB de Mello Malta F Maruta CW Criado PR Castilho VLP do Nascimento Gonçalves EM. Characterization of subtypes of Blastocystis sp. Isolated from patients with urticaria, São Paulo, Brazil. Parasite Epidemiol Control. (2019) 7:e00124. 10.1016/j.parepi.2019.e00124 31872093 PMC6911935

[58] Ascuña-Durand K Salazar-Sánchez RS Castillo-Neyra R Ballón-Echegaray J. Relative frequency of blastocystis subtypes 1, 2, and 3 in urban and periurban human populations of arequipa, peru. Trop Med Infect Dis. (2020) 5:178. 10.3390/tropicalmed5040178 33261137 PMC7709661

[59] Fayer R Santin M Macarisin D. Detection of concurrent infection of dairy cattle with blastocystis, cryptosporidium, giardia, and enterocytozoon by molecular and microscopic methods. Parasitol Res. (2012) 111:1349–55. 10.1007/s00436-012-2971-1 22710524

[60] Angelici MC Nardis C Scarpelli R Ade P. Blastocystis hominis transmission by non-potable water: a case report in Italy. New Microbiol. (2018) 41:173–7. 29498738

[61] Quílez J Sánchez-Acedo C Clavel A Causapé AC. Occurrence of blastocystis sp. In cattle in Aragón, northeastern Spain. Parasitol Res. (1995) 81:703–5. 10.1007/BF00931851 8570589

[62] Rivera WL Tan MAV. Molecular characterization of blastocystis isolates in the philippines by riboprinting. Parasitol Res. (2005) 96:253–7. 10.1007/s00436-005-1344-4 15886995

[63] Barbosa CV de Jesus Batista R Igreja RP Levy CMDA de Macedo HW. Distribution of Blastocystis subtypes isolated from humans from an urban community in Rio de Janeiro, Brazil. Parasit Vect. (2017) 10:1–9. 10.1186/s13071-017-2458-0 29070053 PMC5657060

[64] Wang W Bielefeldt-Ohmann H Traub RJ Cuttell L Owen H. Location and pathogenic potential of blastocystis in the porcine intestine. PLoS ONE. (2014) 9:e103962. 10.1371/journal.pone.0103962 25093578 PMC4122384

[65] Wang J Gong B Yang F Zhang W Zheng Y Liu A. Subtype distribution and genetic characterizations of blastocystis in pigs, cattle, sheep and goats in northeastern China's Heilongjiang Province. Infect Genet Evol. (2018) 57:171–6. 10.1016/j.meegid.2017.11.026 29196130

[66] AbuOdeh R Ezzedine S Madkour M Stensvold CR Samie A Nasrallah G. Molecular subtyping of Blastocystis from diverse animals in the United Arab Emirates. Protist. (2019) 170:125679. 10.1016/j.protis.2019.125679 31580985

[67] Li WC Wang K Gu Y. Occurrence of blastocystis sp. And Pentatrichomonas hominis in sheep and goats in China. Parasit Vect. (2018) 11:1–7. 10.1186/s13071-018-2671-5 29454366 PMC5816562

[68] Tan TC Tan PC Sharma R Sugnaseelan S Suresh KG. Genetic diversity of caprine blastocystis from peninsular Malaysia. Parasitol Res. (2013) 112:85–9. 10.1007/s00436-012-3107-3 22961236

[69] Udonsom R Prasertbun R Mahittikorn A Mori H Changbunjong T Komalamisra C. Blastocystis infection and subtype distribution in humans, cattle, goats, and pigs in central and western Thailand. Infect Genet Evol. (2018) 65:107–11. 10.1016/j.meegid.2018.07.007 30003970

[70] Stenzel DJ Cassidy MF Boreham PFL. Morphology of blastocystis sp. Isolated from circus animals. Int J Parasitol. (1993) 23:685–7. 10.1016/0020-7519(93)90179-3 8225774

[71] Thathaisong U Worapong J Mungthin M Tan-Ariya P Viputtigul K Sudatis A. Blastocystis isolates from a pig and a horse are closely related to blastocystis hominis. J Clin Microbiol. (2003) 41:967–75. 10.1128/JCM.41.3.967-975.2003 12624017 PMC150316

[72] Wang W Cuttell L Bielefeldt-Ohmann H Inpankaew T Owen H Traub RJ. Diversity of blastocystis subtypes in dogs in different geographical settings. Parasit Vect. (2013) 6:1–5. 10.1186/1756-3305-6-215 23883734 PMC3734043

[73] Mohammadpour I Bozorg-Ghalati F Gazzonis AL Manfredi MT Motazedian MH Mohammadpour N. First molecular subtyping and phylogeny of blastocystis sp. Isolated from domestic and synanthropic animals (dogs, cats and brown rats) in southern Iran. Parasit Vect. (2020) 13:1–11. 10.1186/s13071-020-04225-9 32698882 PMC7374852

[74] Nagel R Cuttell L Stensvold CR Mills PC Bielefeldt-Ohmann H Traub RJ. Blastocystis subtypes in symptomatic and asymptomatic family members and pets and response to therapy. Intern Med J. (2012) 42:1187–95. 10.1111/j.1445-5994.2011.02626.x 22032439

[75] Li TS Zou Y Ma YT Ma YY Chen H Liang XX. Molecular characterization of eimeria spp. And Blastocystis in rabbits in Shandong Province, China. Parasitol Res. (2020) 119:1–5. 10.1007/s00436-020-06652-0 32198626

[76] Yoshikawa H Abe N Wu Z. PCR-based identification of zoonotic isolates of blastocystis from mammals and birds. Microbiology. (2004) 150:1147–51. 10.1099/mic.0.26899-0 15133074

[77] Parkar U Traub RJ Kumar S Mungthin M Vitali S Leelayoova S. Direct characterization of blastocystis from faeces by PCR and evidence of zoonotic potential. Parasitology. (2007) 134:359–67. 10.1017/S0031182006001582 17052374

[78] Dogruman-Al F Kustimur S Yoshikawa H Tuncer C Simsek Z Tanyuksel M. Blastocystis subtypes in irritable bowel syndrome and inflammatory bowel disease in Ankara, Turkey. Mem Inst Oswaldo Cruz. (2009) 104:724–7. 10.1590/S0074-02762009000500011 19820833

[79] Moosavi A Haghighi A Mojarad EN Zayeri F Alebouyeh M Khazan H. Genetic variability of blastocystis sp. Isolated from symptomatic and asymptomatic individuals in Iran. Parasitol Res. (2012) 111:2311–5. 10.1007/s00436-012-3085-5 22948205

[80] Stensvold CR Alfellani M Clark CG. Levels of genetic diversity vary dramatically between blastocystis subtypes. Infect Genet Evol. (2012) 12:263–73. 10.1016/j.meegid.2011.11.002 22116021

[81] Alfellani MA Stensvold CR Vidal-Lapiedra A Onuoha ESU Fagbenro-Beyioku AF Clark CG. Variable geographic distribution of Blastocystis subtypes and its potential implications. Acta Trop. (2013) 126:11–18. 10.1016/j.actatropica.2012.12.011 23290980

[82] Russini V Di Filippo MM Fanelli R Polidori M Berrilli F Di Cave D. Characterization of prevalence and genetic subtypes of blastocystis sp. In wild and domestic Suidae of central Italy aided by amplicon NGS. Vet Parasitol Reg Stud Reports. (2020) 22:100472. 10.1016/j.vprsr.2020.100472 33308752

[83] Morris A Robinson G Swain MT Chalmers RM. Direct sequencing of cryptosporidium in stool samples for public health. Front Public Health. (2019) 7:360. 10.3389/fpubh.2019.00360 31921734 PMC6917613

[84] Samie A Tanih NF Seisa I Seheri M Mphahlele J ElBakri A. Prevalence and genetic characterization of Giardia lamblia in relation to diarrhea in Limpopo and Gauteng provinces, South Africa. Parasite Epidemiol Control. (2020) 9:e00140. 10.1016/j.parepi.2020.e00140 32083192 PMC7016452

[85] Maiguashca Sánchez J Sueto SOB Schwabl P Grijalva MJ Llewellyn MS Costales JA. Remarkable genetic diversity of trypanosoma cruzi and trypanosoma rangeli in two localities of southern ecuador identified via deep sequencing of mini-exon gene amplicons. Parasit Vect. (2020) 13:1–13. 10.1186/s13071-020-04079-1 32410645 PMC7227245

[86] Arias-Giraldo LM Muñoz M Hernández C Herrera G Velásquez-Ortiz N Cantillo-Barraza O. Identification of blood-feeding sources in panstrongylus, psammolestes, rhodnius and triatoma using amplicon-based next-generation sequencing. Parasit Vect. (2020) 13:1–14. 10.1186/s13071-020-04310-z 32867816 PMC7457505

[87] Early AM Daniels RF Farrell TM Grimsby J Volkman SK Wirth DF. Detection of low-density plasmodium falciparum infections using amplicon deep sequencing. Malar J. (2019) 18:1–13. 10.1186/s12936-019-2856-1 31262308 PMC6604269

[88] Maloney JG Molokin A Santin M. Assessment of next generation amplicon sequencing of the beta-giardin gene for the detection of Giardia duodenalis assemblages and mixed infections. Food Waterborne Parasitol. (2020) 21:e00098. 10.1016/j.fawpar.2020.e00098 33294649 PMC7691155

[89] Greige S El Safadi D Khaled S Gantois N Baydoun M Chemaly M. First report on the prevalence and subtype distribution of blastocystis sp. In dairy cattle in Lebanon and assessment of zoonotic transmission. Acta tropica. (2019) 194:23–29. 10.1016/j.actatropica.2019.02.013 30878470

[90] Noël C Dufernez F Gerbod D Edgcomb VP Delgado-Viscogliosi P Ho LC. Molecular phylogenies of blastocystis isolates from different hosts: implications for genetic diversity, identification of species, and zoonosis. J Clin Microbiol. (2005) 43:348–55. 10.1128/JCM.43.1.348-355.2005 15634993 PMC540115

