# Supplementary material for: Identification of Multiple Blastocystis Subtypes in Domestic Animals From Colombia Using Amplicon-Based Next Generation Sequencing
Source: Front Vet Sci. 2021 Aug 24;8:732129. doi: 10.3389/fvets.2021.732129 (PMC8421793; doi:10.3389/fvets.2021.732129)
Supplement: Supplementary Figure 1 — Geographic locations of regions in which samples were collected. Departments of Colombia sampled are indicated in the color blue. Orange circles indicate the exact locations of sampling areas. In the legend on the right, the cities/municipalities sampled in each department are shown. [file Data_Sheet_1.PDF]

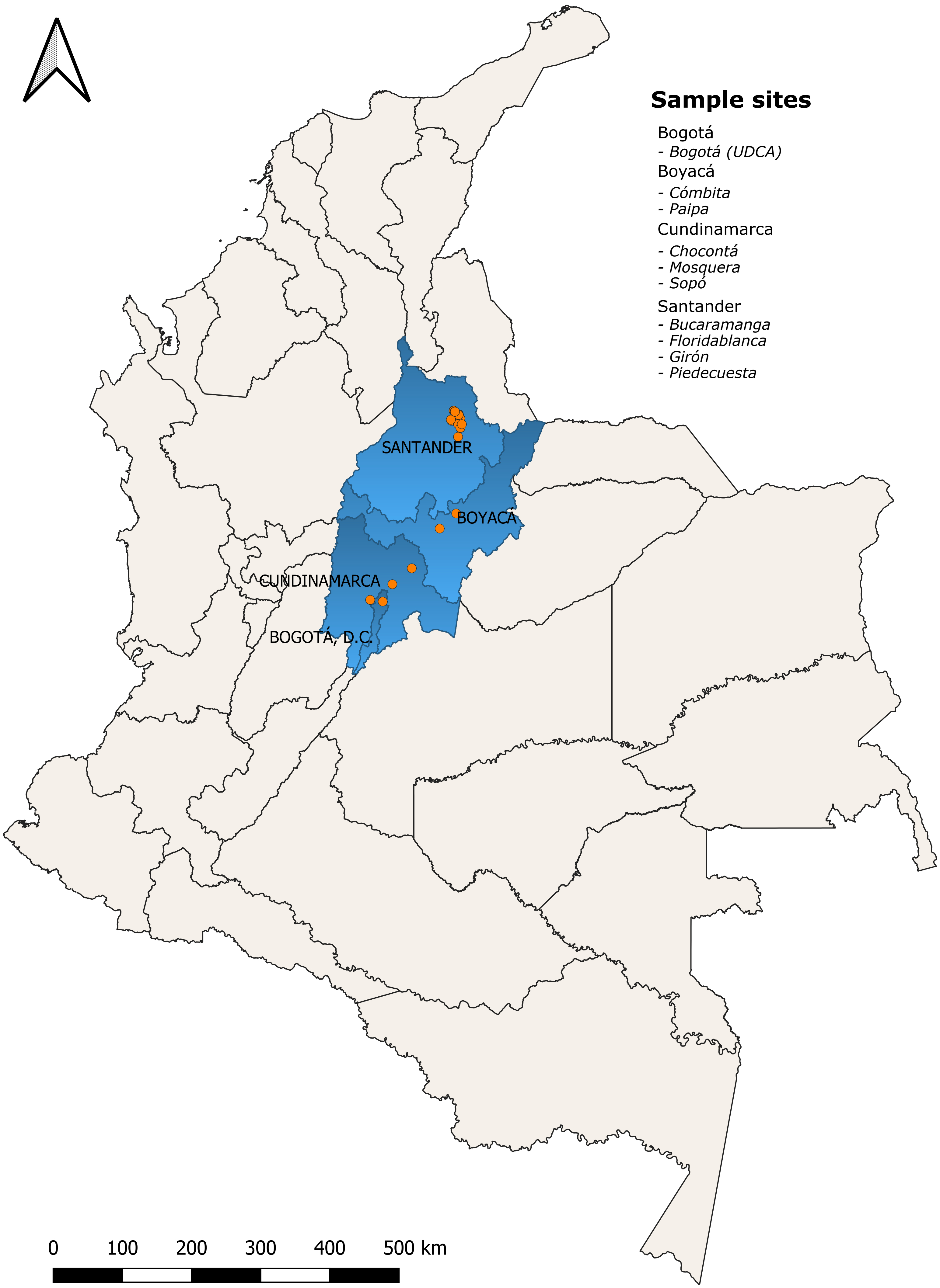

Sample sites
Bogotá
- Bogotá (UDCA)
Boyacá
- Cómbita
- Paipa
Cundinamarca
- Chocontá
- Mosquera
- Sopó
Santander
- Bucaramanga
- Floridablanca
- Girón
- Piedecuesta
SANTANDER
BOYACA
CUNDINAMARCA
BOGOTÁ, D.C.
0
100
200
300
400
500 km
